# Damage Analysis and a Novel Mathematical Relation Between the Interface Quality and the Impact Fracture Energy for Epoxy Composites Reinforced with Medium and High Ramie Woven Fabric Volume Fractions

**DOI:** 10.3390/polym17152105

**Published:** 2025-07-31

**Authors:** Marcelo Vitor Ferreira Machado, Felipe Perissé Duarte Lopes, Noan Tonini Simonassi, Eduardo Atem de Carvalho, Carlos Maurício Fontes Vieira, Sergio Neves Monteiro

**Affiliations:** 1 Mechanical Engineering Department, Fluminense Federal Institute (IFF) and Nucleus of Studies in Applied Thermomechanics, Campos dos Goytacazes 28030-130, RJ, Brazil; 2 Advanced Materials Laboratory (LAMAV), Materials Science Department, State University of Northern Rio de Janeiro (UENF), Campos dos Goytacazes 28013-602, RJ, Brazil; felipeperisse@gmail.com (F.P.D.L.); eatem@uenf.br (E.A.d.C.); vieira@uenf.br (C.M.F.V.); snevesmonteiro@gmail.com (S.N.M.); 3 Materials Science Program, Military Institute of Engineering, Rio de Janeiro 22290-270, RJ, Brazil

**Keywords:** damage analysis, natural fiber polymer composite, interface quality, impact fracture energy

## Abstract

A literature review about polymer composites reveals that natural fibers have been widely used as a reinforcement phase in recent years. In this framework, the lignocellulosic fibers have received marked attention because of their environmental, thermomechanical, and economic advantages for many industrial sectors. This research aims to identify the impact behavior of ramie reinforced epoxy composites with medium- and high-volume fractions of fibers in intact (nonaged) and aged conditions as well as to analyze if the influence of interface quality on the impact fracture energy can be described by a novel mathematical model. To reach these objectives, the study is designed with three groups (40%, 50%, and 60% of fiber theoretical volume fractions) of intact specimens and three groups of aged samples by condensation and ultraviolet radiation (C-UV) simulation containing the same fiber percentages. Consecutively, impact strength and fracture surface analyses are done to expand the comprehension of the damage mechanisms suffered by the biocomposites and to support the development of the mathematical relation. Certainly, this novel model can contribute to more sustainable and greener industries in the near future.

## 1. Introduction

In recent decades, polymer composites reinforced with natural fibers have been widely studied due to their eco-friendly and sustainable characteristics. Additionally, advantages like abundant resources, low acquisition cost, and suitable thermomechanical properties make them even more attractive for automotive, energy, aerospace, and civil construction sectors [1,2,3,4,5,6,7,8,9,10,11,12,13,14].

The replacement of some traditional materials used in different industries by biocomposites can make them more competitive, sustainable, and greener [15,16,17,18]. Nevertheless, in various applications, the impact loadings are critical for the structural safety of many components; therefore, it is essential to study the impact behavior and its consequences on these multiphase materials.

A literature review of natural fiber-reinforced polymer composites presents a representative lack of research regarding the mechanical behavior of these multiphase materials with medium- and high-volume fractions. At the same time, it easily presents that the influence of interface quality on the mechanical performance and, consequently, on the fracture behavior of laminates is a well-established fact [18,19,20,21].

**Figure 1:**
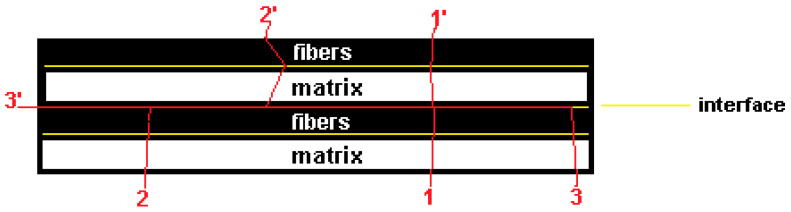
Three independent stationary fracture processes: the intralaminar (path 1-1’), the combined fracture (path 2-2’), and the interlaminar cracking mechanism (path 3-3’).

Basically, the intralaminar fracture consists of matrix and fiber consecutive ruptures; however, between these two composite phases, there is an interface. For that reason, the failure mechanism can be stated as a matrix–interface–fiber successive rupture (path 1-1’, Figure 1). Differently, the interlaminar fractures are characterized by delaminations (path 3-3’, Figure 1), in other words, an interlayer separation. In general, a combination of these failure mechanisms can occur in a laminate when it is subjected to a loading (path 2-2’, Figure 1) [22,23].

In conformity with all this context, the objectives of this research are to characterize the impact behavior of ramie woven fabric-reinforced epoxy composite with medium- and high-volume fractions in intact and C-UV aged conditions and to investigate whether a novel mathematical relation is able to represent the influence of the interface quality on the sort of cracking process and its impact fracture energy.

In summary, the methodology of this research uses the experimental data of the performed Charpy tests and the pertinent descriptive and inferential statistical analyses. In addition, fracture surface characterizations, the laws of classical thermodynamics, the concept of Hamilton’s principle, and literature data are used to obtain the results and to support the study’s conclusions.

Ultimately, the novel relation obtained describes how the interface quality influences the kind of fracture phenomenon and the impact fracture energy of the tested biocomposites. Obviously, this fact can hold up technological developments in view of more sustainable and greener engineering components.

## 2. Materials and Methods

### 2.1. Materials

The polymer used as matrix in this study was a bi-component epoxy resin diglycidyl ether of bisphenol A (DGEBA) with the hardener triethylenetetramine (TETA), both commercially obtained from Epoxyfiber (Rio de Janeiro, Brazil) under the trade names MD 130 and FD 129, respectively. Both DGEBA and TETA are viscous liquids at room temperature that harden when mixed at a mass ratio of 1:0.13 DGEBA:TETA after 4 h, with a complete cure after 7 days. The ramie woven fabric used was purchased as a finalized product.

### 2.2. Production of Biocomposite Plates

The first process was to determine the density of ramie fiber in the acquired woven fabric using 4 pycnometers based on ISO 4787 [24]. A total of 10 measurements were made in each pycnometer, and the obtained mean density of 1.495 g/cm^3^ is in congruence with literature values, for example, as determined by others [25]. That result was later used to determine the number of ramie woven fabric layers for 40%, 50%, and 60% of fiber theoretical volume fractions (see Table 1) according to the rule of mixture and the volume of the metallic mold used. Focusing on the study about impact behavior of epoxy composites with medium- and high-volume fractions of ramie fibers, this research avoided to work below 40% (very explored in the specific literature) and above 60% (tendency to produce very low adhesion for matrix–fiber interfaces in many parts of composite due to the low amount of resin and hard difficulties to manufacture them).

The ramie woven fabric layers placed in a stove for 24 h at 60° was made to remove moisture from them. The mold was coated with vaseline (demolding agent), and the DGEBA:TETA mixture that was done manually during 3 min was added together with the ramie woven fabric layers divided in three steps. In sequence, a slowly hydraulic compression process was made until 6000 kgf/cm^2^ (corrected during 20 min) to shape the plates. In previous works, the cited final pressure was found to be optimal to manufacture composites relatively free of air bubbles. Finally, 24 h after this process, the composite plates were removed from the mold. For this study, we considered six plates: three (40%, 50%, and 60% of fiber theoretical volume fractions) for intact specimens and three for C-UV aged specimens using the same reinforcement percentages, as can be seen in the next section.

### 2.3. Impact Tests and Statistical Analyses

**Figure 2:**
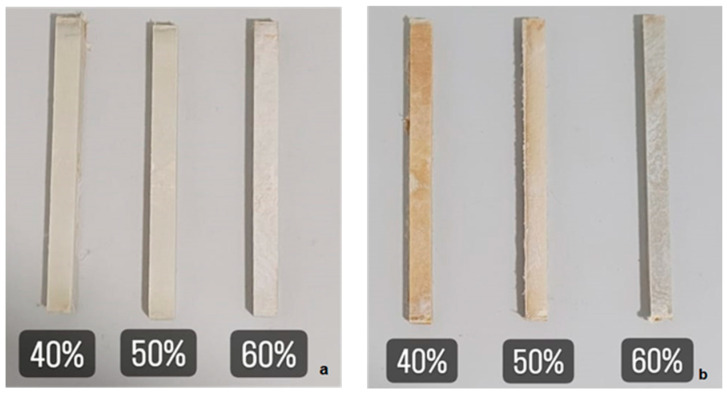
(**a**) Intact specimens and (**b**) aged specimens used in Charpy tests [27].

The standardization ISO 179-1 [26] does not specify temperature or humidity conditions for the Charpy test; however, the room conditions for the test were 16 °C ± 1 °C and 50% RH ± 5% RH. Based on this standardization, the impact test was performed with intact and C-UV aged specimens (Figure 2) using a universal impact test machine PANTEC model X-50C (São Paulo, Brazil) with a 25 J hammer. Specifically, the experiments have been designed with the three groups of composites (40%, 50%, and 60%), both with and without C-UV aging process [27].

As part of experimental measurements, the uncertainties for impact energy, notch length, and area under the notch were 0.05 J, 0.5 mm, and 59.8 mm^2^, respectively. The uncertainty propagations for notch toughness and impact strength of all groups are listed in Table 2. As will be seen from Table 3 and Table 4, the uncertainties presented in Table 2 are entirely covered by the respective standard deviations.

For the C-UV aging process, a COMEXIM accelerated aging machine model ADEXIM was used that operates according to ASTM-G53/154 [28]. The manufacturer’s calibration was made using UVC lamps (100–280 nm) for a temperature range (ambient −90 ± 2 °C). The simulation conditions have been set up based on ASTM 5208 [29]. The aged settings, in conformity with this standardization, were: 504 h of exposure at 70 °C in a cycle of 8 h. 

The descriptive statistics of experimental data and graphs were made using Origin 6.0 software, and the inferential analysis was performed in PAST 4.03 computational tool. Since the Shapiro–Wilk normality test did not confirm a normal distribution for the linear regression residuals of the impact strength data, the one-way ANOVA could not be used because of its homoscedasticity assumption. In this context, the non-parametric Kruskal–Wallis test was performed as a robust alternative analysis to avoid statistical errors (e.g., type I error) [30,31]. Coherently, Dunn’s test was developed to make the pairwise multiple comparisons among impact strength distributions of the groups. In the end, the epsilon-squared and the eta-squared measures for effect size were calculated to obtain the magnitude of the inferential conclusions [32,33].

### 2.4. Intralaminar and Interlaminar Fracture Characterizations

Some typical fracture surface characterizations were obtained using the scanning electron microscope (SEM) Shimadzu SSX550 (Kyoto, Japan) with 0.018 Pa for the vacuum condition. Firstly, a marked intralaminar fracture surface of the intact specimen number 2 (40% group) was analyzed at 45×, 100×, 120× and 300× magnification. In addition, a delaminated surface region of the intact specimen number 8 (60% group) has been observed at 20×, 36×, 100×, and 300× magnification. These characterizations are relevant for the research because they reveal some specific differences between intralaminar and interlaminar damage mechanisms according to the strong and weak matrix–fiber adhesion on fracture surface regions (high or low interface quality), as shall be seen further.

It is important to note that, although the intralaminar and interlaminar characterizations were made from two different fracture surface regions, all the other specimens presenting regions with the same explicit failure mechanisms will reveal the same kind of features.

### 2.5. Hypotheses for the Relation Between the Quality of Interface and Fracture Energy

**Figure 3:**
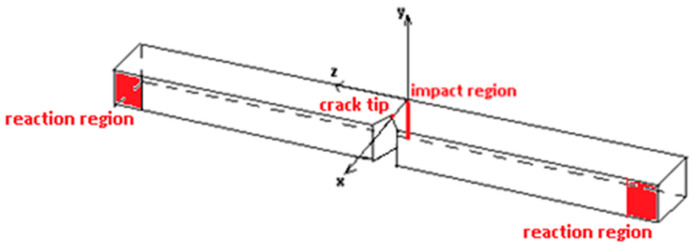
Sketch of a Charpy test specimen with relevant regions in red.

In the Charpy test, due to the impact of hammer on the marked region in Figure 3, immediate reactions in the support regions contribute to the bending of the specimen. An important observation is that ramie woven fabric layers are stacked in parallel to the yz plane of this sketch.

**Figure 4:**
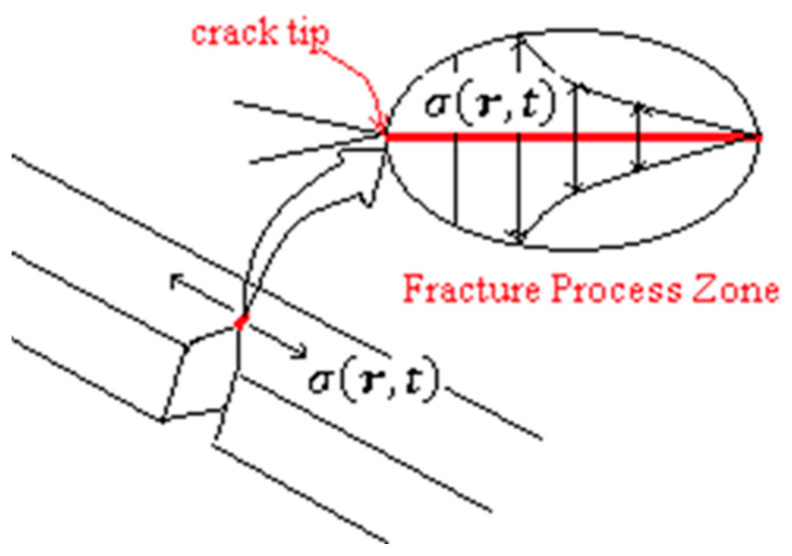
Propagation of tensile stress field due to the impact load during the Charpy test.

The tensile stress caused by the impact loading is propagated in the specimen, leading to the dynamic opening of the notch and successive crack propagation from its tip, as indicated in Figure 4.

As indicated in Figure 4, this stress field is a function of space and time, where **r** = **r**(x,y,z) is the position vector of any point inside the fracture process zone [34,35,36,37,38,39,40,41,42,43]. The crack propagation occurs from the stress concentration initial point, the notch tip. This initial position is **r_0_,** and according to the geometric path done by the crack tip during the fracture process, the final position vector can be determined.

Based on Hamilton’s principle, it can be written that among all possible paths to the crack tip, it will develop that path in which the action is stationary, in other words, as the crack has its evolution during a given time interval (Δt = t_2_ − t_1_), the geometric path developed by the crack tip must be the one that consumes the least energy among all the possibilities for the fracture process.

**Figure 5:**
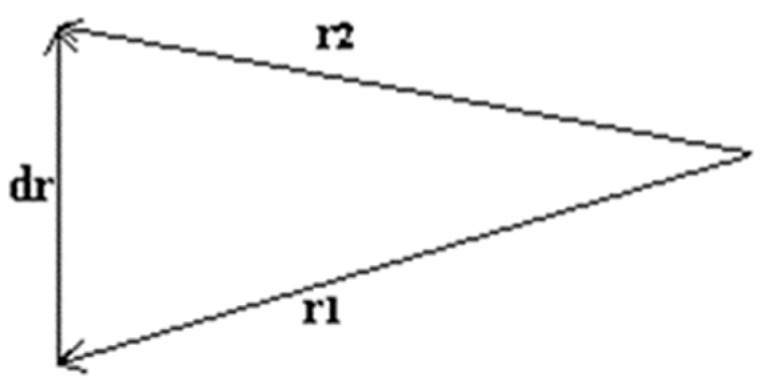
The infinitesimal displacement vector during a continuous deformation.

The least-absorbed energy by a specimen during a fracture process at any point and any instant has an upper limit, which is the necessary energy to maintain the continuous deformation in the material, which can be written as

(1) $$E\left(r,t\right)=\int \left(\int \sigma \left(r,t\right)ndA\right).dr$$

where *dA* is the oriented-area element where the stress field acts and **dr** is the infinitesimal displacement vector of the considered material point, see Figure 5.

Evidently, Equation (1) is based on continuum hypothesis and will present limitations concerning the presence of microcracks and lack of adhesion on matrix–fiber interface regions, i.e., any kind of discontinuity in the laminate. Another question is that $\sigma \left(r,t\right)$ will present jumps in phase transition regions; thus, the integral in Equation (1) must be done step by step in domains where the properties have a smooth continuity.

Considering the presence of all possible discontinuities in material and irreversibility during the dynamic process, the absorbed energy due to fracture is lower than $E\left(r,t\right)$ according to the first and second laws of thermodynamics. This study assumes that impact-absorbed energy in the fracture process can be written on the left-hand side of the expression below:

(2) $$W_{intra}+W_{inter}<E\left(r,t\right)$$

where $W_{intra}$ and $W_{inter}$ are the works of the intralaminar and interlaminar fractures on the damaged region of the laminate, respectively.

Regarding the specific literature, the fiber-reinforced polymer composites present, in general, a quasi-brittle fracture behavior with multiple mechanisms of failure. Intralaminar and interlaminar processes, as well as combinations between them, must be considered in fracture process zone to improve the predictions about these damage phenomena in laminates [44,45,46,47,48,49,50,51,52,53,54,55].

Aiming to describe a relation between the absorbed energy in dynamic fracture processes and the matrix–fiber interface quality of the manufactured biocomposites, this research proposes the definition of a dimensionless variable such as

(3) 0≤ξ≤1

where ξ represents a mean value for the interface quality in a fracture region.

By definition, the value 0 refers to the lowest quality (weakest adhesion between continuous and dispersed phases), and 1 represents the highest interface quality (matrix–fiber excellent adhesion). In this context, when ξ → 1, there is a fracture stationary process similar to the path 1-1’ (Figure 1), and when ξ → 0, the fracture stationary path is similar to 3-3’. The variable representing the interface quality will be introduced in the general inequality (2) in accordance with the analyses of the fracture processes observed in the samples.

## 3. Results and Discussion

### 3.1. Results of the Charpy Test in Intact Specimens

**Figure 6:**
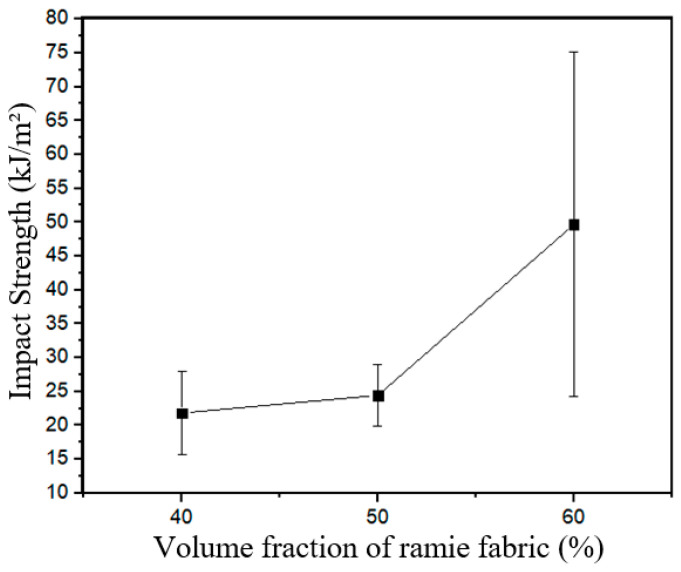
Charpy impact strength vs. fiber volume fractions for the intact specimens.

For the intact specimens with 40%, 50%, and 60% of theoretical volume fractions, the means of energy(J), notch toughness (J/m), and impact strength (kJ/m^2^) can be seen from Table 3. The specific trend analysis relating the impact strength and the theoretical volume fractions of fibers is shown in Figure 6.

An observation of Table 3 and Figure 6 revealed a disproportionate standard deviation for the 60% theoretical volume fraction specimens. This phenomenon can be explained by the significant variation of epoxy amount from some regions to others in those composite specimens since it was a hard manual task due to the 105 ramie woven fabric layers used in that laminate. In other words, the manufacturing difficulties lead to a substantial variation in the interface quality between matrix and fiber, resulting in a marked variance for the 60% intact specimens. As an author’s suggestion, more samples must be used with this fiber volume fraction (60%) in future studies.

**Figure 7:**
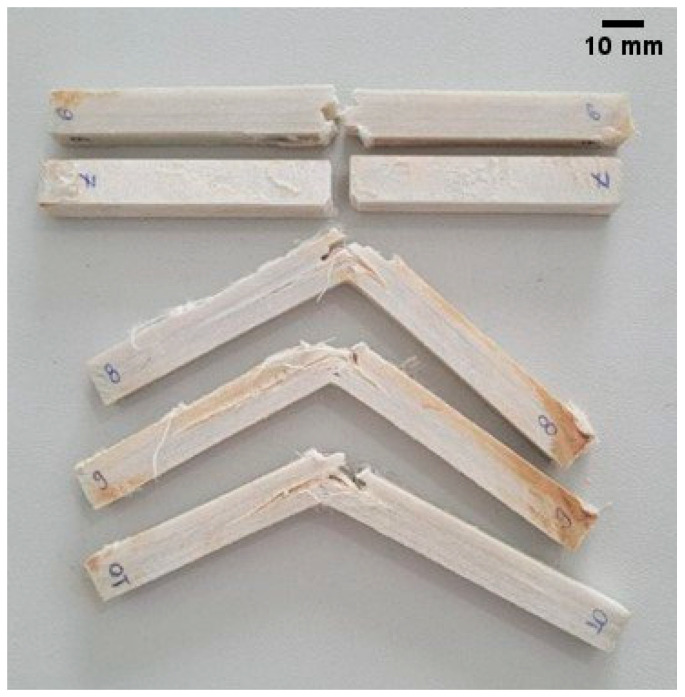
Some fracture surface profiles of intact specimens with 60% of theoretical volume fraction.

The idea discussed in the last paragraph is illustrated in Figure 7. From that illustration can be perceived some different fracture surface profiles. For example, the specimen number 7 has a classical brittle fracture. It presented one of the smallest fracture surfaces; consequently, it absorbed a very low amount of impact energy among the specimens with the highest volume fraction of fibers. In a practical evaluation, the specimen number 7 had only intralaminar fractures. For other specimens in the same figure can be observed delaminated regions on fracture surfaces.

Interlaminar fractures can increase the crack tip path, and in general, the greater the fracture surface, the higher the absorbed impact energy. The exception is when the damage presents, practically, a single delamination process (stationary path 3-3’, seen from Figure 1).

### 3.2. Results of the Charpy Test in Aged Specimens

**Figure 8:**
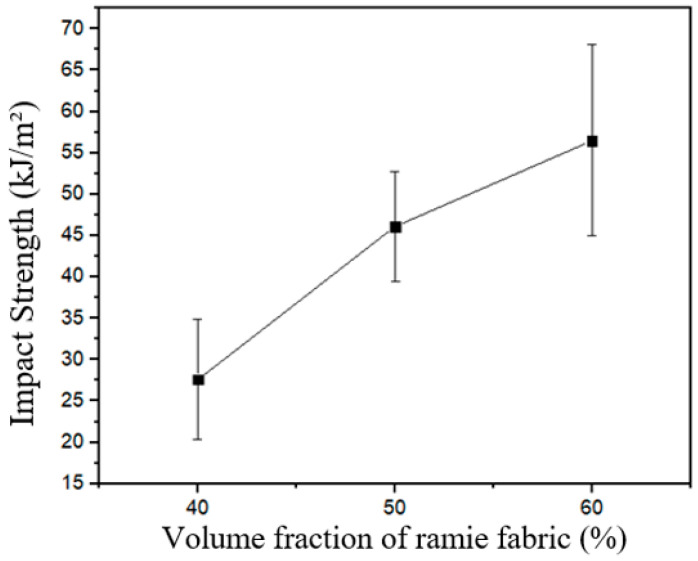
Means of impact strength vs. fiber volume fractions for the C-UV aged specimens.

In Table 4 and Figure 8, it can be seen the means and standard deviations for the results of impact tests with the three groups of C-UV aged specimens. The first notable information from these data is a general growing tendency of the impact strength with the elevation of fiber volume fraction, as also could be observed (Figure 6) for the intact specimens according to another behavior, evidently.

The *p*-value (0.0001463) obtained for the Shapiro–Wilk normality test revealed that the linear regression residuals of impact strength data considering all experimental groups cannot be interpreted according to a Gaussian distribution. For this reason, a Kruskal–Wallis inferential analysis was done and presented the existence of statistical differences among impact strength distributions (*p*-value = 3.793 × 10^−7^). The H statistics obtained for this non-parametric analysis was equal to 37.98. From Table 5, it can be seen that the *p*-values are determined by the subsequent application of Dunn’s post hoc test. The groups with statistical equality among the sampling distributions are highlighted in light blue, and the other pairwise comparisons have statistical distinctions among their impact strength distributions.

To quantify the statistical equalities and differences among the sampling distributions observed in Table 5, the epsilon-squared and eta-squared measures were calculated, and their results (ε^2^ = 0.64 > 0.14 and η^2^ = 0.61 > 0.14) confirm the substantial importance of the inferential characteristics obtained among the groups.

In summary, the early inferential analyses and their large effect size clarify a general trend of impact strength elevation when the reinforcement volume fraction is increased, specifically when there is an increase from 40% to 60% for intact samples and from 40% to 50% for aged ones. These behaviors revealed that there is no necessity for a great increase in fiber volume fraction when the biocomposite is post-cured. This effect occurred probably due to the improvement of the fiber hydrophobicity, and the oxidation process developed when the composite plates were exposed to the heat at 70 °C during aging simulation [56,57,58]. However, the elevation in impact strength tends to a stabilization between 50% and 60% in this case.

### 3.3. The Relation Between Interface Quality and Impact Fracture Energy

**Figure 9:**
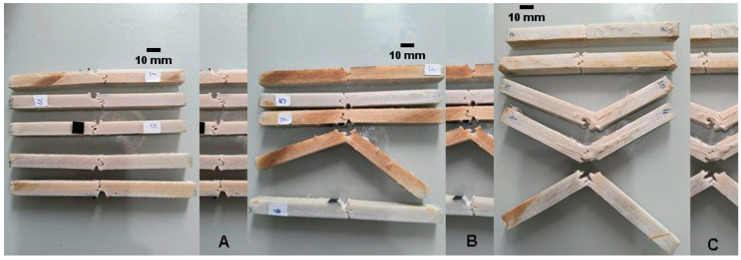
Some fracture profiles in intact specimens: (**A**) 40%, (**B**) 50%, and (**C**) 60% fiber theoretical volume fraction.

To insert a variable representing the interface quality in the general inequality (2), it is necessary to analyze some fracture profiles. Firstly, to investigate how the adhesion level between matrix and fiber affects the development of intralaminar and/or interlaminar fractures, some ruptured nonaged specimens were analyzed (Figure 9).

As can be seen from Figure 9, the majority of fracture profiles presented a significant bumpy surface, alternating short delaminations with the intralaminar failure mechanisms. Additionally, SEM characterizations of some specific regions on fracture surfaces support the analyses of this study.

**Figure 10:**
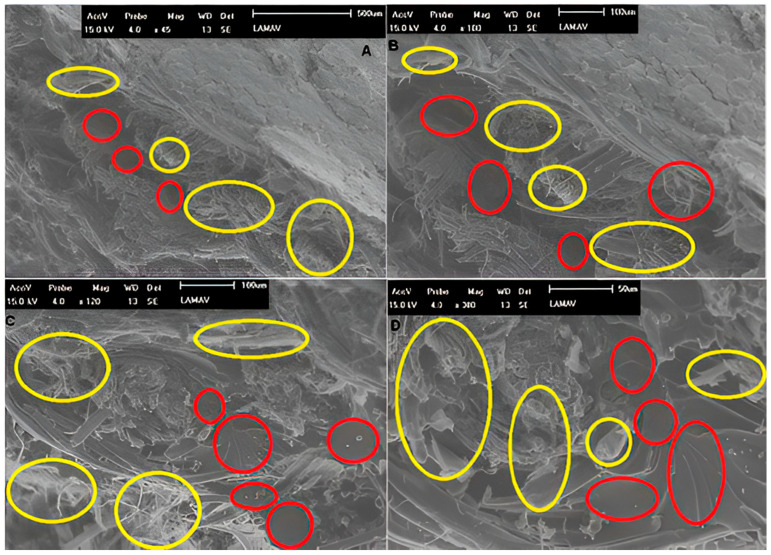
Characterization of a fracture surface region with only intralaminar fracture. All images were made using 15.0 kV. The respective magnifications and scales are (**A**) 45× and 500 μm, (**B**) 100× and 100 μm, (**C**) 120× and 100 μm, and (**D**) 300× and 50 μm.

**Figure 11:**
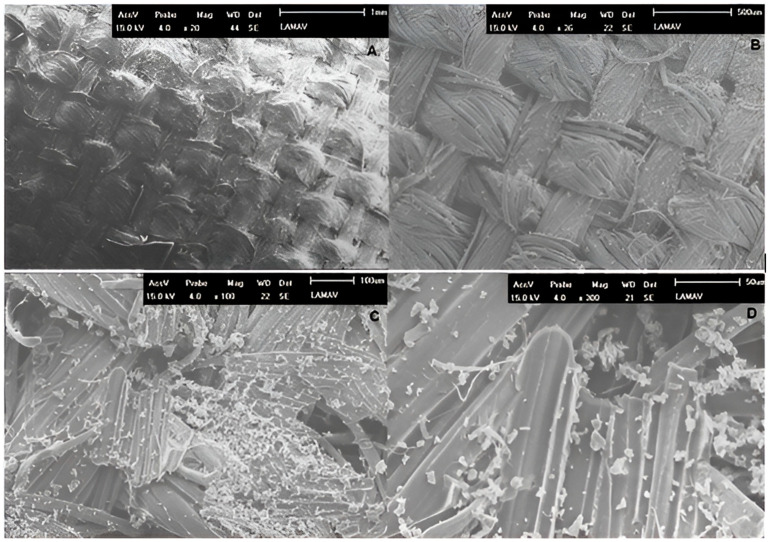
Characterization of a fracture surface with a marked delaminated region. All images were made using 15.0 kV. The respective magnifications and scales are (**A**) 20× and 1mm, (**B**) 36× and 500 μm, (**C**) 100× and 100 μm, and (**D**) 300× and 50 μm.

The fractographic features of the two marked regions indicate that the crack tip took the intralaminar stationary path (Figure 10), and in another case, the fracture was developed according to an interlaminar separation (Figure 11). For the first situation, the region analyzed is characterized by successive ruptures of matrix and fibers through the composite layers. Due to a strong adhesion between the continuous and dispersed phases, there is no layer separation on that fracture surface. Explicitly can be seen ruptures in fibers (circled in yellow) and matrix (smooth and straight parts circled in red) (Figure 10).

The second case (Figure 11) reveals a delaminated region on the fracture surface caused by very weak adhesion between matrix and reinforcement. The layers were separated in this situation due to a poor distribution of epoxy resin (white particulate material) upon the ramie woven fabric (in gray) (see Figure 11).

**Figure 12:**
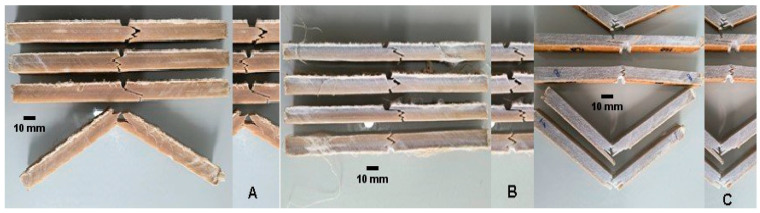
Some fracture profiles of C-UV aged specimens with moderate interface quality: (**A**) 40%, (**B**) 50%, and (**C**) 60% fiber theoretical volume fraction.

In general, for the C-UV aged samples, the fracture behavior of the ramie reinforced epoxy composite with medium and high percentages of fibers was the same as that observed for the intact ones. These specimens presented a fracture surface with successive combinations between intralaminar and interlaminar failures, but in detail, specimens only with a straight cracking surface (brittle fracture), as illustrated in Figure 10, and with a marked delamination process, as presented in Figure 11, were not observed for the aged groups. Hence, a moderate interface quality was easier to achieve for the aged specimens (Figure 12) in comparison to intact ones as a consequence of the post-cure effect stimulated by the ultraviolet radiation exposure.

As a result of the observations done through the Charpy tests with the produced ramie reinforced epoxy composites, the inequality (4) can be proposed to describe how the interface quality is associated with each sort of fracture and how the general quasi-brittle behavior, due to presence of multiple discontinuities in the laminate, limits the value of absorbed energy in comparison with the continuum model.

From the novel mathematical relation between interface quality and the impact fracture energy proposed in this research (inequality 4), it is trivial to realize that we have ξ = 1 for the highest interface quality and ξ = 0 for the lowest one. For these two fracture processes represented by the extreme values of ξ (purely intralaminar and widely interlaminar, respectively), it could be verified some smallest and largest cracking areas, but always with low absorbed energy and low impact strength.

Otherwise, when the fracture process is a combination between short intralaminar and interlaminar ruptures, there are cracking surfaces with intermediary areas and impact strength elevations observed. In other words, a moderate adhesion between matrix and the ramie woven fabric is preferable and adequate for impact strength and energy absorption.

(4) $$\xi W_{intra}+{(1-\xi )W}_{inter}<E\left(r,t\right)$$

## 4. Conclusions

In accordance with the inferential analyses, the elevation of impact strength is marked between 40% and 50% of fiber volume fraction for aged samples. Nevertheless, there is no statistical difference from 50% to 60%, i.e., when the aged specimens were subjected to 70 °C before the Charpy test, there is no necessity for a great rise in the reinforcement volume fraction to elevate the impact strength. It is relevant to note that the post-cure effect influenced significantly specimens with fiber volume fraction over 40%. The exposure, on the other hand, only a considerable elevation from 40% to 60% in fiber volume fraction, is required to produce a statistical raise of impact strength for intact specimens.

Concerning the fracture characterizations, some intact specimens presented a straight and smooth cracking surface, which indicates an accentuated brittle fracture influenced by excellent adhesion of matrix–fiber interfaces. Other few samples revealed wide delamination regions on the fracture surfaces due to their very weak adhesion between matrix and reinforcement (low interface quality). Differently, the aged specimens did not present these two extreme features for the fracture surfaces when subjected to the impact tests. This fact presents a general tendency of interface quality concentration around the defined mean value of ξ (0.5) due to the post-cure effect for the C-UV aged specimens.

Satisfactorily, the novel mathematical relation proposed (inequality 4) represents the influence of the interface quality on intralaminar and/or interlaminar fracture mechanisms and, consequently, on the impact fracture energy. In conformity with the studies of the specific literature [59,60,61,62,63,64], the model proposed here is groundbreaker in describing mathematically a direct relation between the interface quality and impact energy. Consequently, it represents an advancement for this applied science field.

Certainly, the model presented in this research can contribute to impact behavior predictions of polymer composites reinforced by natural fibers and to complement other novel models in future studies. In addition, this research also supports directly a gradual insertion of these sustainable and eco-friendly composite materials in several industrial sectors.

## Figures and Tables

**Table 1:** Fiber theoretical volume fractions used and the respective number of ramie woven fabric layers.

Fiber Theoretical Volume Fraction	40%	50%	60%
Number of layers	70	87	105

**Table 2:** Uncertainty propagation for notch toughness and impact strength of all groups.

	Δ (Notch Toughness) (J/m)	Δ (Impact Strength) (kJ/m^2^)
40% int.	±9.36	±0.89
50% int.	±10.14	±0.87
60% int.	±23.13	±0.87
40% aged	±13.52	±0.68
50% aged	±19.90	±0.85
60% aged	±25.23	±0.91

**Table 3:** Means and standard deviations of Charpy tests using intact specimens with 40%, 50%, and 60% of fiber theoretical volume fractions.

%Vol_fiber_	Energy (J)	Notch Toughness (J/m)	Impact Strength (kJ/m^2^)
40%	1.28 ± 0.35	139.91 ± 37.67	21.83 ± 6.08
50%	1.48 ± 0.36	158.53 ± 36.10	24.45 ± 4.59
60%	3.86 ± 1.87	414.09 ± 200.24	49.67 ± 25.44

**Table 4:** Means and standard deviation of Charpy tests using C-UV aged specimens with 40%, 50%, and 60% of fiber theoretical volume fractions.

%Vol_fiber_	Energy (J)	Notch Toughness (J/m)	Impact Strength (kJ/m^2^)
40%	2.31 ± 0.77	231.11 ± 76.60	27.62 ± 7.25
50%	3.54 ± 0.61	354.40 ± 61.12	46.11 ± 6.59
60%	4.55 ± 1.18	455.00 ± 118.22	56.47 ± 11.55

**Table 5:** The *p*-values of Dunn’s post hoc test for the impact strength data for the intact (int.) and aged composites.

	40% Int.	50% Int.	60% Int.	40% Aged	50% Aged	60% Aged
40% int.		0.4621	0.0003	0.1971	9.6 × 10^−5^	1.4 × 10^−6^
50% int.	0.4621		0.0047	0.5603	0.0017	5.1 × 10^−5^
60% int.	0.0003	0.0047		0.0340	0.6992	0.2031
40% aged	0.1971	0.5603	0.0340		0.0146	0.0009
50% aged	9.6 × 10^−5^	0.0017	0.6992	0.0146		0.3876
60% aged	1.4 × 10^−6^	5.1 × 10^−5^	0.2031	0.0009	0.3876	

## Data Availability

Data are contained within the article.
